# Intermittent hypoxic perconditioning improves cognitive function in a mouse model of vascular cognitive impairment and dementia with comorbidities by recovering cerebral blood flow

**DOI:** 10.4103/NRR.NRR-D-24-00716

**Published:** 2025-01-29

**Authors:** Feiyang Jin, Zhengming Tian, Yuying Guan, Yuning Li, Yakun Gu, Mengyuan Guo, Qianqian Shao, Yingxia Liu, Xiuhai Guo, Zhenzhen Quan, Jia Liu, Xunming Ji

**Affiliations:** 1 Beijing Institute of Brain Disorders, Laboratory of Brain Disorders, Laboratory for Hypoxia Adaptation Translational Medicine, Ministry of Science and Technology, Collaborative Innovation Center for Brain Disorders, Beijing Advanced Innovation Center for Big Data-Based Precision Medicine, Capital Medical University, Beijing, China; 2 Department of Neurology, Xuanwu Hospital, Capital Medical University, Beijing, China; 3 Key Laboratory of Molecular Medicine and Biotherapy, School of Life Science, Beijing Institute of Technology, Beijing, China; 4 Department of Neurosurgery, Xuanwu Hospital, Capital Medical University, Beijing, China

**Keywords:** bilateral carotid artery stenosis, cerebrovascular microcirculation, chronic cerebral hypoperfusion, cognitive function, high fat-high fructose diet, hippocampus, intermittent hypoxia, mitochondrial respiratory chain, prefrontal cortex, vascular cognitive impairment and dementia

## Abstract

Vascular cognitive impairment and dementia is a debilitating neurological disorder caused by chronic cerebral hypoperfusion, for which no effective causative treatments are currently available. Intermittent hypoxia has been shown to enhance cerebral blood flow in mice, but its efficacy in a model of vascular cognitive impairment and dementia remains unclear. In this study, we established a mouse model of vascular cognitive impairment and dementia by bilateral carotid artery stenosis. Intermittent hypoxia was induced before and after this stenosis. We found that intermittent hypoxia increased cerebral blood flow, oxygen saturation, and microcirculation in the prefrontal cortex and hippocampus in the model mice, without causing neurovascular damage. Additionally, intermittent hypoxia significantly improved cognitive function in the mouse model of vascular cognitive impairment and dementia, with perconditioning showing greater efficacy than preconditioning. Improvements in cerebral microcirculation and blood flow were positively correlated with cognitive recovery. Even in a mouse model of vascular cognitive impairment and dementia with comorbidities induced by a high-fat, high-fructose diet, intermittent hypoxic perconditioning demonstrated protective effects on cognitive function. Proteomic analysis indicated that mitochondrial protection is a key mechanism, particularly through upregulating NDUFB8 expression and increasing the activity of mitochondrial complex I. These findings suggest that intermittent hypoxia is a potential non-invasive strategy for the prevention and treatment of vascular cognitive impairment and dementia.

## Graphical abstract

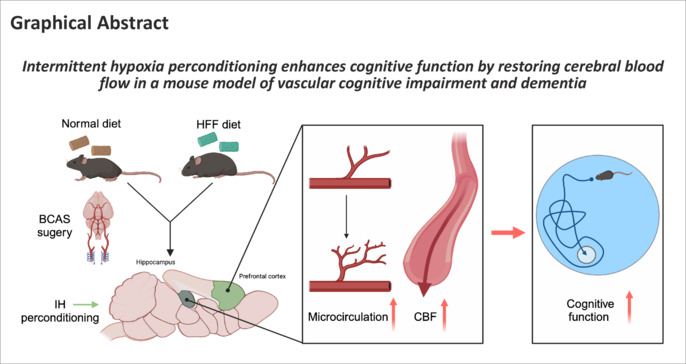

## Introduction

Vascular cognitive impairment and dementia (VCID) is a heterogeneous cognitive impairment syndrome, predominantly affecting older adults, caused by cerebrovascular dysfunction and the ensuing cerebral blood flow (CBF) insufficiency (O’Brien and Thomas, 2015; Corriveau et al., 2016; van der Flier et al., 2018). The wide extent of this CBF insufficiency negatively impacts multiple cognitive domains, including memory and executive function. Vascular dementia, the end stage of vascular cognitive impairment, results in a marked decline in daily living abilities, social functioning, and quality of life (Skrobot et al., 2018). Currently, VCID is the second leading cause of dementia worldwide, following Alzheimer’s disease, imposing significant caregiving and economic burdens on families and societies (Jorm and Jolley, 1998; Lobo et al., 2000). Treatment strategies for VCID focus mainly on controlling potential vascular risk factors and associated symptoms because there are no targeted treatments for the underlying causes (Moretti et al., 2011; Godyń et al., 2016; Wolters and Ikram, 2019; Sinha et al., 2020). As the principal pathogenic mechanism for VCID, chronic cerebral hypoperfusion is a promising therapeutic target for VCID (Dichgans and Leys, 2017; Poh et al., 2022). Our preliminary research has shown that intermittent hypoxia (IH) can effectively promote angiogenesis and enhance CBF in mice (Guan et al., 2023b). However, whether this intervention is effective in mouse models of VCID remains unclear.

To critically evaluate the preventive and therapeutic efficacy of IH for VCID, an appropriate animal model should be selected. Bilateral common carotid artery stenosis (BCAS) surgery is the most frequently used method to construct VCID mouse models because it effectively induces chronic cerebral hypoperfusion and closely recapitulates the progression of VCID (Washida et al., 2019). Because of progressive development of cerebral hypoperfusion and ensuing neural damage, patients with VCID often present with substantial and medically intractable cognitive impairment at the time of diagnosis. Therefore, the timing of IH intervention is a critical factor in restorative efficacy. Furthermore, as a disorder primarily impacting older adults, VCID is frequently comorbid with known age-related risk factors for vascular impairment such as hypertension, diabetes, and obesity. These conditions interact synergistically with the primary pathology to trigger the onset and exacerbate the progression of VCID. However, most basic studies on VCID pathomechanisms have employed adult male mice receiving BCAS alone, which does not fully simulate VCID with comorbid risk factors. This limitation may explain the low clinical translation success rate of various VCID prevention and treatment strategies (Fisher and Stroke Therapy Academic Industry Roundtable, 2003; Fisher et al., 2009; Kilkenny et al., 2010; Duncombe et al., 2017; Hainsworth et al., 2017).

In this study, we investigate the preventive and therapeutic effects of IH in a VCID mouse model, particularly focusing on the impact of comorbid conditions on treatment efficacy. Additionally, we explore the potential molecular mechanisms underlying the protective effects of IH.

## Methods

### Animals

Male C57BL/6J mice, aged 6–8 weeks and weighing (18–25) g, were purchased from SpePharm Biotechnology Co., Ltd. (license No. SCXK (Jing) 2019-0010; Beijing, China). In total, 165 mice of specific pathogen-free grade were housed under controlled temperature (22 ± 1°C) and humidity (50% ± 5%) conditions, with a 12-hour/12-hour light/dark cycle (lights on at 8 a.m.) and *ad libitum* access to food and water prior to the experiments. All animal housing and experimental procedures were approved by the Animal Care and Use Committee of Capital Medical University (approval No. AEEI-2024-096; April 19, 2024) and performed according to the National Institutes of Health Guide for the Care and Use of Laboratory Animals. All efforts were made to minimize animal suffering and the number of animals used. All animal experiments complied with the ARRIVE guidelines (Percie du Sert et al., 2020).

Mice were initially randomly divided into groups on the basis of research stage (e.g., control and IH groups). All mice in the respective groups participated in experiments aimed at detecting CBF and oxygen saturation and cerebral vascular permeability, and in behavioral tests and metabolic assessments. However, for experiments involving fluorescence staining and small-animal magnetic resonance imaging, four to six mice per group were randomly selected to undergo these procedures because of time and economic constraints.

### Intermittent hypoxia treatment

Mice were placed in gas-controlled chambers and exposed to 10 cycles of 5-minute normoxia (21% O_2_) and 5-minute hypoxia (13% O_2_) every day for 14 consecutive days. Control mice were placed in the same gas-controlled chambers for the same period, but with normal air circulating continuously (Guan et al., 2023b). All mice had free access to food and water during treatment. Based on different intervention time points, we divided intermittent hypoxia into preoperative IH preconditioning and postoperative perconditioning (meaning IH intervention during disease progression).

### Detection of cerebral blood flow and oxygen saturation

We used the MoorVMS vascular monitoring system, with laser Doppler perfusion and a temperature monitor (Moor Instruments Ltd., Axminster, UK), to measure cortical perfusion and oxygen saturation. Briefly, the midline of the parietal lobe was disinfected, and a longitudinal incision was made to fully expose the skull. To ensure the reliability of the results, five points were randomly measured around the sensorimotor cortex for the analysis. The specific location is 1.0–2.5 mm posterior to the bregma and 2.0–3.0 mm lateral to the midline (Franklin and Paxinos, 2007).

### Cerebral vascular permeability

Blood vessel integrity was assessed by measuring the permeability to sodium fluorescein. Briefly, 30 minutes following injection of sodium fluorescein (F6377-100G, 2%, 5 mL/kg; Sigma-Aldrich, St. Louis, MO, USA), mouse brains were excised, divided into individual regions, and homogenized separately. Fluorescein leakage into the parenchyma was estimated by measuring 520-nm emission from 485-nm excitation on a CLARIOstar fluorescence microplate reader (BMG LABTECH, Offenburg, Germany). Relative permeability in each region was calculated by dividing the fluorescence emission measured in IH-treated mouse brain lysate by that measured in control mouse brain lysate.

### Nissl staining

Mice treated as indicated were sacrificed to evaluate neural injury arising from the intervention. They were anesthetized via intraperitoneal injection of 10% pentobarbital (P3761; Sigma-Aldrich) at a dose of 0.12 mL/kg, and deep anesthesia was confirmed by the absence of reflexes. Then, the chest cavity was opened, and a needle was inserted into the left ventricle for transcardial perfusion. The mice were first perfused with 20 mL of cold saline to flush out the blood, followed by 20 mL of freshly prepared cold 4% paraformaldehyde for tissue fixation. The brains were then removed, fixed in 4% paraformaldehyde, dehydrated by incubation in a descending alcohol gradient, embedded in wax, trimmed, and sectioned into 4-μm coronal slices. Slices were dewaxed by two incubations in xylene for 20 minutes each, dehydrated by successive incubation in 100% ethanol, 90% ethanol, and 75% ethanol for 5 minutes each, and then rinsed with tap water. Finally, the slices were stained with Nissl dye (G1036; Servicebio, Wuhan, China) for 3–5 minutes, immersed in 0.1% glacial acetic acid differentiation solution, and sealed with neutral gum for examination under light microscopy (3D HISTECH, Budapest, Hungary) as described below (Yang et al., 2024).

### Cortical tissue clearing and imaging

As previously described, cortical tissue clearing was performed to clear the whole-brain tissue (Zhao et al., 2021). Briefly, brain tissue was post-fixed in 4% paraformaldehyde for 4 days and then incubated in 0.1 M phosphate-buffered saline (pH 7.5) containing 8% sodium dodecyl sulfate (L3771; Sigma-Aldrich) at 37°C under 100 r/min shaking until the tissue was clear; the clearing solution was replaced every few days. After clearing, tissues were washed in 0.01% phosphate-buffered saline plus Tween-20 for 24 hours at 37°C with 100 r/min shaking, and incubated in Focus Clear (CelExplorer Labs, Taipei, Taiwan, China) for 2 hours. Cleared sections were photographed under light-sheet microscopy (Light-sheet Z.1; Zeiss, Oberkochen, Germany), with the Z-stack set at 2.5 μm and an X–Y scanning resolution of 1800 × 1800 pixels. Images were stitched into a series of whole-brain transverse images and exported in raw format to Imaris software (version 10.1.0; Bitplane, Zurich, Switzerland) for three-dimensional (3D) reconstruction.

### Immunofluorescence staining of microvasculature

Lectin (DL-1178-1, 1:100; 100 μL/25 g; Vector Lab, Taipei, Taiwan, China) was injected via the tail vein 30 minutes before heart perfusion with saline and 4% paraformaldehyde. Brains were removed, dehydrated in a gradient of 20% and 30% sucrose, and cut into 100-μm-thick coronal sections using a freezing microtome (Leica, Wetzlar, Germany). Sections were then sealed immediately and photographed using a fluorescence confocal microscope (TCS SP8; Leica).

### Three-dimensional rendering and quantification

The image processing and quantification methods applied here were described previously (Zhao et al., 2021). Briefly, light-sheet microscopy and confocal images were imported into Imaris software and assembled into 3D images using the filament and surface tools in the Imaris software. The source channel was selected, and an appropriate threshold was set manually to differentiate the target signal (stain) from the background. Non-specific signals were then removed using the filter tool. Voxels inside the surface were set to 100 in the mask channel of the surface tool. Then, 3D-rendered images were constructed. All analyses were conducted using the statistics function of the filament tool. The reconstructed vasculature was then observed by the surface tool.

### Surgical procedure for bilateral common carotid artery stenosis

Anesthesia was rapidly induced by 5% isoflurane (RWD Life Science Co., Ltd., Shenzhen, China) and maintained under 1.5% isoflurane using a ventilator (RWD Life Science Co., Ltd.). BCAS surgery was conducted as described previously (Nishio et al., 2010; An et al., 2021). In brief, the bilateral common carotid arteries were exposed individually and ligated together by coils with an inner diameter of 0.18 mm, pitch of 0.50 mm, and total length of 2.5 mm. Body temperature was maintained at 36.5–37.5°C during surgery using a heating pad and feedback from a rectal thermometer. To confirm stenosis, CBF was monitored using laser speckle flowgraphy (RWD Life Sciences Co., Ltd.). In the sham group, the bilateral common carotid arteries were exposed but not ligated by coils. Cognitive function was evaluated after 2 or 4 weeks of recovery.

### Novel object recognition test

The novel object recognition (NOR) test was conducted as described previously (Lueptow, 2017; Bu et al., 2024). For 3 consecutive days, each mouse was placed in an open testing chamber and allowed to explore for 10 minutes. After this habituation phase, mice were placed in the same chamber, now containing two identical objects placed about 10 cm from the chamber wall and 15 cm apart. The time spent interacting (sniffing, pawing) with these two objects and the latency to interaction were recorded. On the following day, one of the objects was replaced with a novel object, and the latency and interaction times were recorded. Shorter latency and additional time spent interacting with the novel object compared with the familiar object are considered to indicate NOR. Mice not reaching a 20-second minimum exploration time for both objects were excluded.

### Morris water maze test

The Morris water maze (MWM) was used to evaluate visuospatial learning and memory, as described previously (Ben-Ari et al., 2019). Briefly, mice were trained to locate a submerged platform in a circular pool (three trials/day over 5 days). The time taken to find the platform (escape latency) was recorded as a measure of spatial learning. If a test mouse did not find the platform within 60 seconds, it was placed on the platform and allowed to remain thereon for 5 seconds. On day 6, the platform was removed, and mice were allowed to swim freely for 60 seconds. Mice repeatedly floating in the water and refusing to swim were excluded from the experiment (Vorhees and Williams, 2006). The number of times that a test mouse crossed the former platform location and the swim pathlength were recorded as measures of spatial memory.

### Magnetic resonance imaging

All magnetic resonance imaging scans were acquired using a Bruker 7-Tesla horizontal bore imaging system (Billerica, MA, USA). Following positioning and pilot scans, T2-weighted images were acquired using a rapid acquisition with relaxation enhancement (RARE) sequence with the following parameters: echo time (TE)/repetition time (TR), 20/4000 ms; number of averages, 8; matrix size, 160 × 160, number of slices, 25; slice thickness, 0.5 mm; RARE factor, 4; and field of view, 16 × 16 mm^2^. Arterial spin labeling was used to measure CBF. Images with different inversion times were acquired using RARE sequences with the following parameters: RARE factor, 72; TE/TR, 46/10,000 ms; number of averages, 1; matrix size, 128 × 128; field of view, 20 × 20 mm^2^; in-plane spatial resolution, 313 × 313 μm^2^; slice thickness, 1.0 mm; number of slices, 1; and inversion times, 30, 100, 200, 300, 400, 500, 600, 700, 800, 900, 1000, 1100, 1200, 1300, 1400, 1500, 1600, 1700, 1800, 1950, 2100, and 2300 ms. The total scan time was 8 minutes 24 seconds. Diffusion tensor imaging scans covering the entire brain, including 5 reference and 30 non-collinear diffusion-weighted images, were also acquired using a multislice spin echo sequence with the following parameters: TE/TR, 22/2800 ms, number of averages, 2; matrix size, 160 × 160; field of view, 16 × 16 mm^2^; number of axial slices, 25; slice thickness, 0.5 mm; b value, 3000 s/mm^2^; and Δ/δ, 11/5 ms.

### High-fat, high-fructose feeding

Mice were fed a high-fat diet consisting of 60% fat, 20% carbohydrates, and 20% protein (D12942; Research Diets, New Brunswick, NJ, USA). Mice were also provided with a 30% fructose solution prepared from fructose supplied by Macklin (D809612; Shanghai, China). The fructose solution was made fresh and changed every 2–3 days. Mice were monitored for general health, weight gain, and blood glucose throughout the study, with detailed records kept to ensure compliance with institutional animal care guidelines. Mouse body weight was measured using an electronic balance (CFC-400C; Weizhixiang, Zhejiang, China) by subtracting the tare weight of the container from the total weight of the mouse and container combined. To measure blood glucose, a small blood sample was collected from the tail vein and applied to a test strip in an electronic glucose meter (GA-3; Sinocare, Guangzhou, China).

### Western blotting

Proteins were extracted from cortical tissues using radioimmunoprecipitation assay buffer (Solarbio, Beijing, China), and total lysate protein concentrations were quantified using the Pierce BCA Protein Assay Kit (Thermo Fisher Scientific, Waltham, MA, USA). Lysate samples were boiled in loading buffer for 10 minutes, and total proteins were separated by sodium dodecyl sulfate-polyacrylamide gel electrophoresis. Separated proteins were then transferred to polyvinylidene fluoride membranes for immunoblotting. Membranes were blocked with 5% skim milk at 20–25°C for 1 hour, washed with Tris-buffered saline containing 0.1% Tween, and incubated overnight at 4°C with NDUFB8 (rabbit, 1:10,000, Cat# 14794-1-AP, RRID: AB_2150970; Proteintech, Chicago, IL, USA), Cox IV (rabbit, 1:10,000, Cat# 11242-1-AP, RRID: AB_2085278; Proteintech), and β-actin (mouse, 1:5000, Cat# EM21002, RRID: AB_2819164; Huaan, Hangzhou, China) primary antibodies. Immunoblotted membranes were washed three times in Tris-buffered saline containing 0.1% Tween and incubated with secondary antibodies (IRDye 680RD Goat Anti-Mouse IgG [H+L; 1:10,000, Cat3 926-68070, RRID: AB_10956588; Licor, Lincoln, NE, USA] and IRDye 800CW Goat Anti-Rabbit IgG [H+L; 1:10,000, Cat3 926-68070, RRID: AB_10956588; Licor]) at 20–25°C for 1 hour. Immunolabeling was detected using Odyssey Infrared Imaging System (Odyssey, Lincoln, NE, USA). Band intensity was measured using ImageJ (1.53a; National Institutes of Health, Bethesda, MD, USA) (Schneider et al., 2012), normalized to that of β-actin (gel loading control), and compared statistically using GraphPad Prism software (version 9.4.1 for Mac OS; GraphPad Software, La Jolla, CA, USA).

### Mitochondrial complex I activity assay

Mouse brain tissues stored at –80°C were suspended in 1 mL of Extraction Buffer I and quickly homogenized on ice using a glass homogenizer. The homogenate was then centrifuged at 4°C, 600 × *g* for 10 minutes, and the supernatant was transferred to a new centrifuge tube for a second centrifugation at 4°C, 11,000 × *g* for 15 minutes. The supernatant was discarded, and the pellet was mixed with 200 μL of Extraction Buffer I and 200 μL of Extraction Buffer II, followed by sonication to obtain the mitochondrial extract. The concentration of the mitochondrial extract obtained via the above steps was quantified using a BCA assay kit (BC0515-100T/96S, Solarbio). Extract samples and working solution were added to a 96-well plate and mixed thoroughly, followed 10 seconds later by measurement of the absorbance (A1) at 340 nm. The plate was promptly placed in a 37°C water bath for 1 minute, removed, and dried quickly, and the absorbance (A2) was measured again after 1 minute 10 seconds. Mitochondrial complex I activity is represented by the change in absorbance at 340 nm per minute, ultimately expressed as the ratio to the sham group.

### Proteomic analysis

We also conducted differential proteome expression analysis to identify cortex-specific effects of IH at the molecular level. The procedure was as follows (Shao et al., 2022): sample preparation, tandem mass tag labeling, peptide identification, and bioinformatic analysis. First, left cortical tissues were separated, homogenized in lysis buffer, and centrifuged twice for 5 minutes at 5000 × *g* and 4°C. The total protein concentration in the supernatant was measured using the Bradford protein assay, and a 200-μg sample was incubated with 5 μL of 200 mM reducing reagent for 1 hour at 55°C. Iodoacetamide (5 μL of 375 mM concentration) was added to the solution, and the mixture was incubated for another 10 minutes. Then, 200 μL of 100 mM dissolution buffer (AB Sciex, Framingham, MA, USA) was added, and the new solution was sequentially centrifuged for 20 minutes at 12,000 × *g* and digested in trypsin for 14 hours at 22°C. Peptide fragments were lyophilized and redissolved in 100 mM dissolution buffer for tandem mass tag labeling. A 100-μg sample was then dissolved in 41 μL of tandem mass tag reagent (90111; Thermo Fisher Scientific) plus 0.8 mg/tube absolute ethyl alcohol. After 1 hour, 5% quenching reagent was added for 15 minutes. Labeled peptides were lyophilized and stored for later analysis by nano ultra-high-performance liquid chromatography–tandem mass spectrometry (MS). Briefly, acquired peptide fractions were suspended in 20 μL of buffer A (0.1% formic acid, 2% acetonitrile) and centrifuged for 10 minutes at 12,000 × *g*. Next, 10 μL of the supernatant was injected into the nano ultra-high-performance liquid chromatography–tandem MS system, consisting of a Nanoflow high-performance liquid chromatography system (EASY-nLC 1000; Thermo Fisher Scientific) equipped with an Acclaim PepMap100 C18 column, EASY-Spray C18 column, and Orbitrap Fusion Lumos mass spectrometer (Thermo Fisher Scientific). The mass spectrometer was operated in positive ion mode (source voltage, 2.1 kV), and full MS scans were performed over the range of 300–1500 m/z at a resolution of 120,000. For MS scans, the 20 most abundant ions with multiple charge states were selected for higher-energy collisional dissociation fragmentation following one full MS scan. The peptide false discovery rate was determined from peptide spectrum matches when searched against the reverse decoy database. Peptides assigned to only one protein group were considered unique. The false discovery rate was set to 0.01 for protein identification.

Peptides were identified by searching against the Uniprot_Mus_musculus (https://www.uniprot.org) database and processed using Proteome Discoverer 1.4 (Thermo Fisher Scientific). The protein identification criteria were as follows: precursor ion mass tolerance, ±15 ppm; fragment ion mass tolerance, ±20 mmu; max missed cleavages, 2; static modification, carboxy aminomethylation (57.021 Da) of Cys residues; and dynamic modifications, oxidation modification (+15.995 Da) of methionine residues. Finally, hierarchical clustering analysis evaluated batch effects in the proteomic data for each sample. Samples within the same treatment group exhibited high similarity, whereas those from different sample groups were obviously different. A protein–protein interaction network was constructed using Cytoscape (version 3.9.1; Cytoscape Consortium, La Jolla, CA, USA). Gene Ontology analysis was performed using Metascape (v3.5; Metascape Consortium, Los Angeles, CA, USA).

### Statistical analysis

The operator was blinded to the experimental groups. All data analyses and quantifications were conducted in a blinded (to the experimental conditions) manner. The Shapiro–Wilk test was used to assess dataset normality before further statistical analyses. Normally distributed data are reported as mean ± standard error of the mean, whereas non-normally distributed variables are presented as median (interquartile range). Two groups with normal variable distributions were compared by the independent-samples *t*-test (two-tailed), and two groups with at least one non-normally distributed variable were compared by the Mann–Whitney *U* test. Multiple groups with normal distributions were compared by one- or two-way analysis of variance (pairwise comparisons), whereas multiple groups with a non-normal distribution were compared by Kruskal–Wallis analysis of variance. The *post hoc* tests used included Dunn’s test, Tukey’s test, and Sidak’s correction. Correlations between two variables were assessed using linear regression analysis. Regarding the statistical reporting, N indicates the number of biological replicates (e.g., individual mice) per experimental group. *P* < 0.05 was considered statistically significant for all tests except that evaluating differential protein expression, where *P* ≤ 0.05 and fold-change ≥ 1.2 or ≤ 0.67 were required for statistical significance. All statistical analyses were performed using GraphPad Software.

## Results

### Intermittent hypoxia enhances microcirculation in the prefrontal cortex and hippocampus without causing neurovascular damage

**Figure 1:**
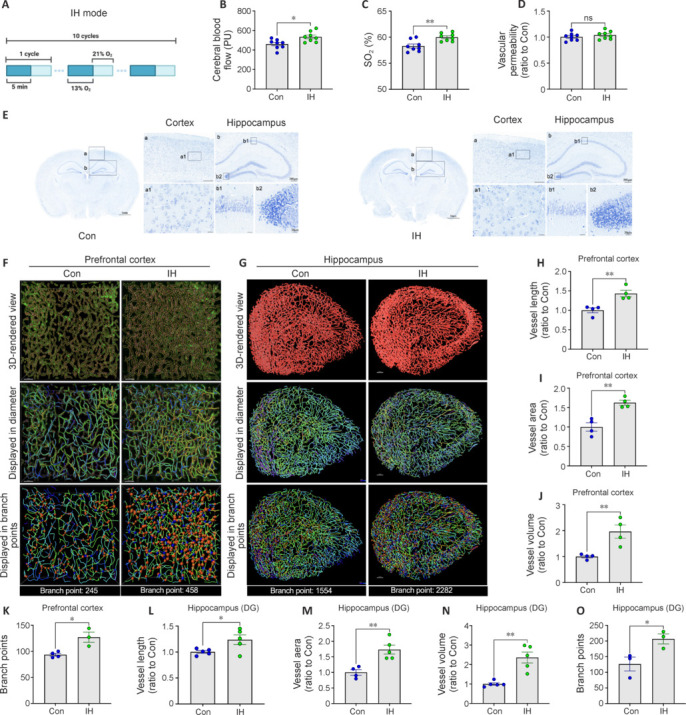
Intermittent hypoxia (IH) enhances microcirculation within the prefrontal cortex (PFC) and hippocampus without inducing neurovascular damage. (A) The IH regimen. (B, C) Cerebral blood flow (CBF; B) and oxygen saturation (SO_2_; C) in mouse cortex after IH and in the control group (*n* = 8 mice per group). (D) Vascular permeability, quantified using the fluorescein leakage method, in control and IH group mice (*n* = 5 or 6 mice per group). (E) Neuropathology in hippocampal and cortical tissues visualized by Nissl staining. No morphological or structural abnormalities were observed in the neurons of the cortex or hippocampus of IH group mice. Scale bar for the control group and IH group: left, 1 mm; right, 200 µm up, 20 µm down. (F, G) Three-dimensional (3D) rendered view of microvessels in the PFC (F, scale bars: 50 μm) and hippocampus (G, scale bars: 100 μm) showing a greater number of branch points in IH mice compared with controls. Different colors represent vessel diameters. (H–O) Quantification showing enhanced vessel length in the PFC (H) and hippocampus (L) (*n* = 4 or 5 mice per group), vessel area in the PFC (I) and hippocampus (M) (*n* = 4 or 5 mice group), vessel volume in the PFC (J) and hippocampus (N) (*n* = 4 or 5 mice per group), and number of branch points in the PFC (K) and hippocampus (O) (*n* = 3 or 4 mice per group) of IH group mice compared with controls. Results are expressed as mean ± standard error of the mean. *P < 0.05, **P < 0.01. Con: Control group; DG; dentate gyrus; IH: intermittent hypoxia group; ns: not significant.

Young adult mice were exposed to 10 5-minute periods of hypoxia (13% O_2_) interspersed with 5 minutes of normoxia (21% O_2_), once daily for 14 consecutives (**Figure 1A**). Compared with the control group, IH significantly increased CBF (*P* = 0.0119) and oxygen saturation (*P* = 0.0030), as measured under normoxia post-treatment in both the prefrontal cortex (PFC) and hippocampus (**Figure 1B** and **C**). Moreover, these increases in brain perfusion and oxygenation were not accompanied by vascular damage, as evidenced by sodium fluorescein leakage measurements (**Figure 1D**), or neurocellular degeneration, as evidenced by Nissl staining (**Figure 1E**), at 14 days post-IH, suggesting that IH can improve brain metabolism without inducing chronic neurovascular damage.

To examine if this effect of IH was related to angiogenesis or alterations in neurovascular structure, we labeled the vascular endothelium by injecting lectin into the tail vein and conducted morphometric analyses, both in situ and in brain slices. To enhance tissue transparency while preserving fluorescent signals, the whole brain and the brain tissue sections were subsequently subjected to a clearing process (**Additional Figure 1A**). Comprehensive examination of cerebral vasculature *in situ*, via tissue clearing and imaging, revealed notable increases in vascular density and the number of branching points within both the PFC and hippocampus of IH-exposed mice compared with sham-treated controls (**Figure 1F** and **G**). In accordance with the findings in intact brains, subsequent quantitative analysis of the microvasculature within brain tissue slices demonstrated marked increases in vessel length, vessel area, vessel volume, and the number of branch points in the PFC (**Figure 1H–K**) and hippocampus (**Figure 1L–O**) compared with sham controls. We also analyzed vascular parameters such as mean vessel diameter, vessel resistance, and vessel straightness, but there were no marked group differences in these basic vascular characteristics (**Additional Figure 1B–G**). Thus, IH enhanced CBF and oxygen saturation by promoting angiogenesis.

### Intermittent hypoxia reduces cognitive impairment in the vascular cognitive impairment and dementia mouse model

**Figure 2:**
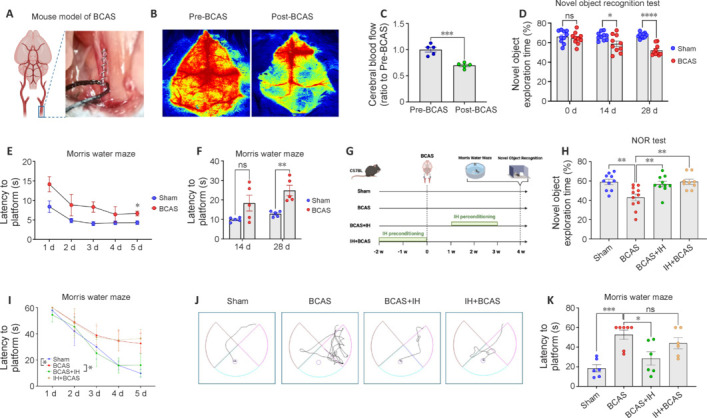
Intermittent hypoxia (IH) mitigates cognitive dysfunction in vascular cognitive impairment and dementia (VCID) model mice. (A) Bilateral common artery stenosis (BCAS) model of VCID. (B, C) Representative images (B) and quantitative analysis (C) of CBF in mice before and after BCAS surgery (*n* = 5 mice per group), as measured by laser speckle. After BCAS surgery, there was a significant decrease in CBF in the mice. (D) Novel object recognition (NOR) test performance was impaired following BCAS (*n* = 10 mice per group). (E) Escape latency in the Morris water maze (MWM) was prolonged in BCAS mice compared with sham surgery controls. Tests were conducted from day 14 to day 19 after BCAS or sham surgery (*n* = 6 mice per group). (F) Mean escape latencies were significantly longer in the BCAS group than in the sham surgery control group on days 14 and 28 post-surgery (*n* = 5 per group). (G) Schema of experiments involving BCAS or sham surgery, and IH before or after surgery. (H) NOR test results on day 28 after BCAS (*n* = 9–11 mice per group) showing that both IH regimens partially reversed cognitive impairment. (I) MWM training performance from day 28 to day 32 after BCAS showing that only IH perconditioning improved spatial learning (*n* = 5 mice per group). (J) Representative swim paths for different groups in the MWM. The path to the platform was shortest for the sham group and longest for the BCAS group. Both IH preconditioning and IH perconditioning reduced the path length to the platform compared with BCAS, with IH perconditioning showing a more pronounced effect. (K) Mean escape latencies on day 33 after BCAS (*n* = 6 or 7 mice per group). All results are expressed as mean ± standard error of the mean. *P < 0.05, **P < 0.01, ***P < 0.001, ****P < 0.0001. BCAS: BCAS group; BCAS + IH: BCAS combined with IH perconditioning group; IH + BCAS: BCAS combined with IH preconditioning group; ns: not significant; sham: sham surgery group.

To investigate the potential protective effect of IH against VCID-associated cognitive dysfunction, we established a BCAS model (**Figure 2A**) and confirmed significantly reduced CBF (*P* = 0.0004) after surgery (**Figure 2B** and **C**) and significantly impaired (*P* < 0.0001) NOR at 28 days post-surgery in these mice compared with sham surgery control mice (**Figure 2D**). Furthermore, escape latency to find the submerged platform on day 5 of MWM training was markedly longer in the BCAS group than the sham surgery control group at 14 days post-surgery (**Figure 2E** and **F**).

We then examined if IH could mitigate cognitive dysfunction in this VCID model: both preconditioning before surgery (IH + VCID group) and perconditioning after surgery (VCID + IH group) increased the time spent exploring the novel object compared with untreated VCID model mice on day 28 post-surgery (**Figure 2G**). Moreover, both IH groups spent about the same amount of time with the novel object as sham surgery control mice (**Figure 2H**). Further, VCID + IH mice took markedly less time to find the submerged platform versus untreated VCID model mice on day 28 post-surgery, consistent with improved spatial learning. However, the IH + VCID group showed no such effect (**Figure 2I–K**). These results suggest perconditioning is more effective for mitigating cognitive dysfunction in VCID model mice than preconditioning.

### Intermittent hypoxia perconditioning enhances cerebral perfusion and microcirculation within cognition-relevant brain regions of vascular cognitive impairment and dementia mice

**Figure 3:**
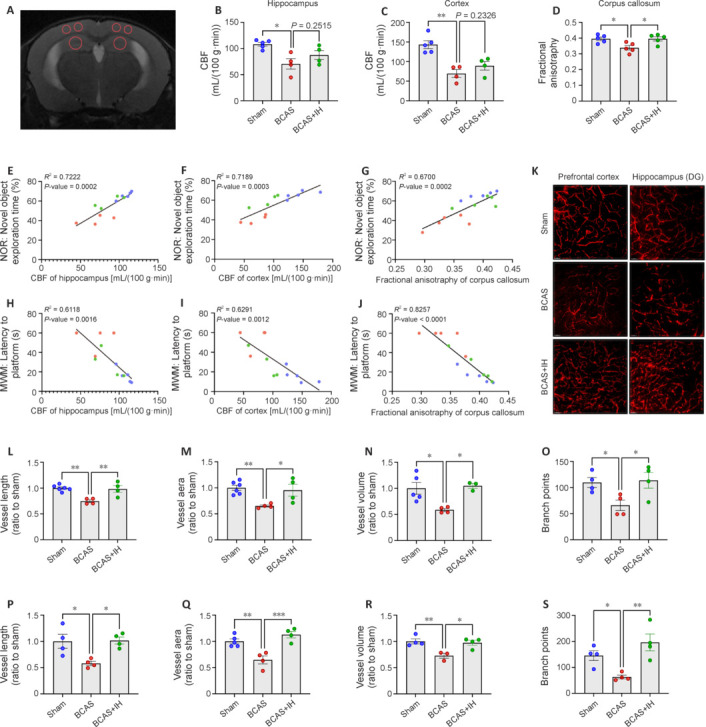
Intermittent hypoxia (IH) after bilateral carotid artery stenosis (BCAS) surgery improves cerebral perfusion and microcirculation within cognition-related brain regions of vascular cognitive impairment and dementia model mice. (A) Sample small-animal magnetic resonance image for measuring cerebral blood flow (CBF). The red circles represent the cortex and hippocampus (from top to bottom). (B) Quantitative results from arterial spin labeling (ASL) sequences showing restoration of CBF in the hippocampus of BCAS mice via IH perconditioning (*n* = 4 or 5 mice per group). (C) Quantitative results from ASL sequences showing restoration of CBF in the prefrontal cortex (PFC) of BCAS mice via IH perconditioning (*n* = 4 or 5 mice per group). (D) Quantitative results from diffusion tensor imaging sequences showing preservation of corpus callosum (CC) white matter integrity in BCAS mice via IH perconditioning. Integrity is expressed on the basis of fractional anisotropy (FA) (*n* = 5 mice per group). (E–J) Linear regression analyses of novel object exploration time in the novel object recognition (NOR) test (E) *versus* hippocampal CBF (*n* = 13 mice group), novel object exploration time *versus* cortical CBF (*n* = 13 mice per group) (F), novel object exploration time *versus* FA of the CC (*n* = 15) (G), escape latency in the Morris water maze (MWM) test *versus* hippocampal CBF *(n* = 13 mice per group) (H), escape latency in the MWM test *versus* cortical CBF (*n* = 13) (I), and escape latency in the MWM test *versus* FA of the CC (*n* = 15 mice per group) (J). The results strongly suggest that angiogenesis mediates cognitive enhancement via IH perconditioning in BCAS model mice. (K) Representative images of blood vessels in the mouse PFC and hippocampal dentate gyrus (DG) area (scale bars: 50 μm). Compared with the sham group, the BCAS group showed reduced microcirculatory density in both the PFC and the hippocampal DG region, whereas the BCAS + IH group exhibited an improvement in microcirculatory density in these regions. (L–S) Quantification showing enhanced vessel length in the PFC (*n* = 4–6 mice per group) (L), vessel area in the PFC (*n* = 4–6 mice per group) (M), vessel volume in the PFC (*n* = 4 or 5 mice per group) (N), number of branch points in the PFC (*n* = 4 mice per group) (O), vessel length in the hippocampus DG (*n* = 4 mice per group) (P), vessel aera in the hippocampus DG (*n* = 4 or 5 mice per group) (Q), vessel volume in the hippocampus DG (*n* = 3 or 4 mice per group) (R), and number of branch points in the hippocampus DG (*n* = 4) (S) of BCAS mice receiving IH perconditioning compared with untreated BCAS mice. All results are expressed as mean ± standard error of the mean. *P < 0.05, ***P < 0.001. BCAS + IH: BCAS combined with IH perconditioning group; sham: sham surgery group.

As IH treatment following BCAS was more effective than preconditioning in ameliorating cognitive impairments in VCID mice, we examined the influence of this treatment regimen on CBF and microcirculation in the PFC and hippocampus. Small-animal magnetic resonance imaging revealed a pronounced reduction in CBF within the hippocampus and PFC of VCID model mice versus the sham surgery group, whereas there was no marked reduction in CBF in the VCID + IH group (**Figure 3A–C**). This suggests IH intervention partially reversed the BCAS surgery-induced reduction in CBF. Furthermore, diffusion tensor imaging revealed damage to corpus callosum fibers in VCID model mice; this damage was also attenuated by IH perconditioning (**Figure 3D**). Linear regression analyses further revealed positive correlations of CBF in the hippocampus and PFC with both corpus callosum white matter integrity and cognitive performance (**Figure 3E–J**). In accordance with these *in situ* results, 3D reconstruction of microvessels in brain slices (**Figure 3K**) revealed enhanced vessel length, vessel area, vessel volume, and the number of branch points in the PFC and hippocampus dentate gyrus region of VCID + IH group mice versus VCID mice (**Figure 3L–S**). We also analyzed basic vascular characteristics, including vessel mean diameter, vessel resistance, and vessel straightness; however, no marked differences in these parameters were found among the three groups (**Additional Figure 2A–F**). Thus, enhanced CBF because of IH treatment following BCAS likely helps mitigate both corpus callosum damage and cognitive dysfunction.

We also performed Nissl staining, Fluoro-Jade B staining (**Additional File 1**), and microglia staining (**Additional File 2**) on mouse brain tissue to observe pathological damage and inflammatory cell infiltration in cognition-related brain regions. IH perconditioning improved the reduction and disorganization of neurons (**Additional Figure 2G**), alleviated neurodegenerative changes (**Additional Figure 2H** and **I**), and reduced the microglial activation and infiltration (**Additional Figure 2J** and **K**) caused by BCAS.

### Intermittent hypoxia perconditioning also enhances cerebral blood flow and improves cognitive function in vascular cognitive impairment and dementia mice with comorbid risk factors

**Figure 4:**
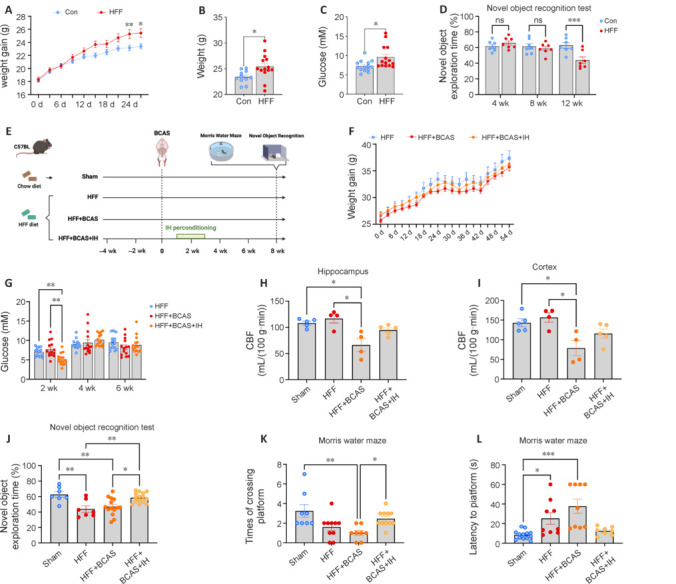
Intermittent hypoxia (IH) following bilateral carotid artery stenosis (BCAS) surgery increases cerebral blood flow (CBF) and enhances cognitive function in vascular cognitive impairment and dementia model mice with concurrent risk factors. (A) Changes in mouse body weight over time (*n* = 12–14 mice per group) during standard and high-fat, high-fructose (HFF) diet feeding. (B) Mouse body weight after 4 weeks of standard and HFF diet feeding (*n* = 11–14 mice per group). (C) Blood glucose levels after 4 weeks of standard and HFF diet feeding (*n* = 14 mice per group). (D) Novel object exploration time in the novel object recognition (NOR) test after 4, 8, and 12 weeks of standard and HFF diet feeding (*n* = 7 mice per group) showing that HFF alone impairs cognition. (E) Schematic of experiments involving BCAS surgery and IH. (F) Changes in mouse body weight over time (*n* = 14 or 15 mice per group). (G) Changes in blood glucose levels over time (*n* = 14 or 15 mice per group). (H) Quantitative results from arterial spin labeling (ASL) sequences showing that IH perconditioning restores hippocampal CBF in BCAS model mice (*n* = 4 or 5 mice per group). (I) Quantitative results from ASL sequences showing that IH perconditioning restores CBF in the prefrontal cortex of BCAS model mice (*n* = 4 or 5 mice per group). (J) Novel object exploration time in the NOR test (*n* = 7–15 mice per group): restoration via IH perconditioning. (K) Number of platform crossings in the Morris water maze (MWM) probe test showing that IH perconditioning preserves spatial memory capacity in BCAS mice (*n* = 8–11 mice group). (L) Escape latency in the MWM showing that IH perconditioning preserves spatial learning capacity in BCAS mice (*n* = 8–13 mice group). All results are expressed as mean ± standard error of the mean. *P < 0.05, **P < 0.01, ***P < 0.001. ns: not significant; Con: control group; HFF + BCAS: HFF diet combined with BCAS group; HFF + BCAS + IH: HFF diet combined with BCAS and IH intervention group; sham: sham surgery group.

Patients with VCID often present with comorbid risk factors such as diabetes, hypertension, and obesity (Wang et al., 2023). To establish a more clinically relevant VCID model, we combined BCAS surgery with high-fat, high-fructose (HFF) feeding. Naive mice maintained on the HFF diet exhibited markedly greater body weight gain starting on day 24 compared with standard diet-fed mice, and after 28 days, HFF diet-fed mice exhibited markedly elevated fasting blood glucose versus the standard diet group (**Figure 4A–C**). More prolonged HFF (12 weeks) also reduced NOR test performance (**Figure 4D**).

We next evaluated the impact of IH on metabolic parameters, CBF, and cognitive function in mice receiving both an HFF diet and BCAS surgery (**Figure 4E**). IH perconditioning failed to attenuate the HFF-diet-induced weight gain (**Figure 4F**). Furthermore, elevated blood glucose levels were only transiently reduced during IH, before reverting to baseline (**Figure 4G**). We also examined blood cell composition (**Additional Figure 3A–H**) and lipid levels (**Additional Figure 3I–L**). Notably, IH perconditioning did not reverse the lipid abnormalities caused by the HFF diet. The HFF diet did not diminish cerebral perfusion in control mice; however, HFF + BCAS group mice exhibited notable CBF reductions in the hippocampus and cortex, which were partially reversed by IH (**Figure 4H** and **I**). Both the HFF-alone and HFF + BCAS groups demonstrated cognitive deficits in the NOR and MWM test following 8 weeks of HFF feeding; these effects were partially mitigated by IH perconditioning (**Figure 4J–L**) despite only marginal changes in metabolic parameters. Moreover, IH perconditioning alleviated the neurodegeneration induced by HFF and BCAS (**Additional Figure 3M** and **N**), aligning with the observed improvement in the cognitive deficits caused by these conditions.

### Intermittent hypoxia induces the differential expression of functionally diverse proteins

**Figure 5:**
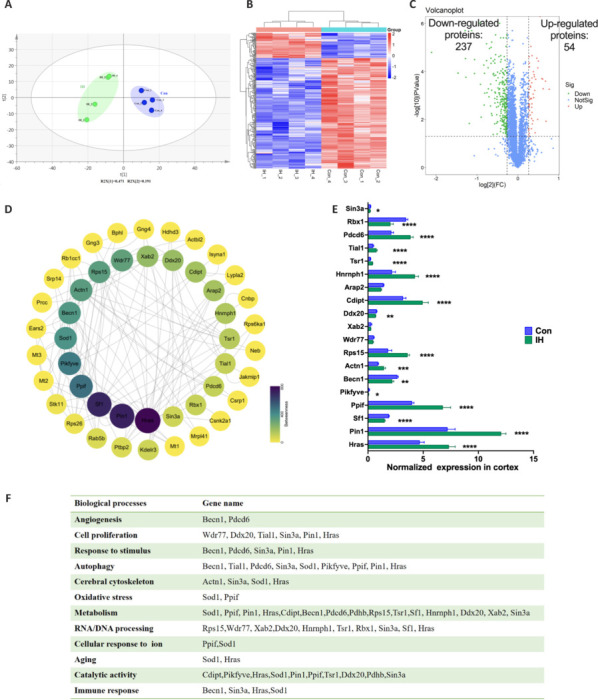
Proteins differentially expressed in the cortex of intermittent hypoxia (IH)-treated mice. (A) Principal component analysis of the whole proteome of IH and control mice. (B) Heat map presenting characteristic concentration profiles for control and IH group mice, with upregulated proteins in red, downregulated proteins in blue, and unchanged proteins in white. (C) Volcano plots highlighting significant differences in protein expression between IH and control mice. (D) Proteins with significant differential expression (fold-change ≥ 1.2 or ≤ 0.67 and *P* ≤ 0.05) in interaction network analysis. (E) Top proteins in the inner circle were statistically analyzed (*n* = 4). Results are expressed as mean ± standard error of the mean. *P < 0.05, **P < 0.01, ***P < 0.001, ****P < 0.0001. (F) Biological processes of top proteins according to Gene Ontology analysis. Con: Control group; IH: intermittent hypoxia group.

To examine the molecular mechanisms mediating the effects of IH on CBF, vascular density, and cognition, we conducted a global proteomics analysis of cortical tissue isolated 14 days post-treatment. A database search using MS spectra from each tandem mass tag sample identified 4826 proteins across all time points, and principal component analysis revealed clear separation between the control and IH groups (**Figure 5A**). The differential expression analysis results are displayed as a heat map and volcano map in **Figures 5B** and **C**, respectively. The 20 proteins with the strongest differential expression (among those with fold-change ≥ 1.2 or ≤ 0.67, *P* ≤ 0.05) were identified using the betweenness centrality function in Cytoscape and used to construct a protein–protein interaction network (**Figure 5D**). Statistical (**Figure 5E**) and Gene Ontology enrichment analyses (**Figure 5F**) revealed “angiogenesis,” “cell proliferation,” and “cerebral cytoskeleton” as the predominant biological processes enriched among differentially expressed proteins such as *Becn1*, *Pdcd6*, *Wdr77*, *Ddx20*, *Tial1*, *Sin3a*, *Pin1*, *Hras*, and *Actn1*. Thus, these proteins may be particularly important in the cerebrovascular growth response to IH. Other enriched biological processes among the differentially expression proteins included “autophagy,” “metabolism,” and “catalytic activity,” suggesting IH also upregulates mitochondrial metabolic capacity.

### Intermittent hypoxia modulates the expression of proteins associated with multiple stages of the mitochondrial respiratory chain

**Figure 6:**
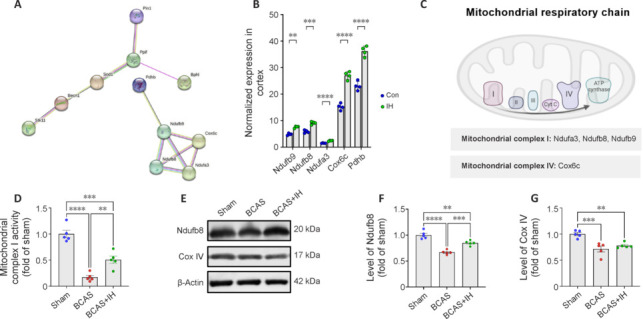
Intermittent hypoxia alters the cortical expression levels of multiple mitochondrial respiratory chain proteins and enhances complex 1 activity. (A) Mitochondrial protein–protein network analysis. (B) Statistical analysis of respiratory chain-related protein expression levels (*n* = 4 mice per group). (C) Respiratory chain complex proteins exhibiting differential expression among groups. (D) Mitochondrial complex Ι activity in the prefrontal cortex (PFC) (*n* = 5 mice per group). (E) Representative immunoblots of Ndufb8 and Cox IV in the PFC. (F, G) Quantification of Ndufb8 (F) Cox IV protein (G) expression levels in the PFC (*n* = 5 mice per group). Results are expressed as mean ± standard error of the mean. **P < 0.01, ***P < 0. 001, ****P < 0.0001. BCAS: BCAS group; BCAS + IH: BCAS combined with IH perconditioning group; Con: control group; IH: IH group; sham: sham surgery group.

Differentially expressed proteins localized to mitochondria were predominantly associated with “catalytic activity” and “respiration” (**Figure 6A**), including respiratory chain-related proteins (**Figure 6B**) such as *Ndufb9*, *Ndufb8*, and *Ndufa3*, localized to mitochondrial complex I, and Cox6c, localized to mitochondrial complex IV (**Figure 6C**). To assess functional relevance, we measured mitochondrial complex I activity in the PFC across groups, finding significantly greater activity (*P* = 0.0057) in BCAS + IH mice than BCAS mice (**Figure 6D**). Moreover, western blotting confirmed that BCAS markedly downregulated Ndufb8 and Cox IV expression in the cortex, and this effect was partially reversed by IH (**Figure 6E–G**). Thus, the improvement in CBF induced by IH may have enhanced mitochondrial metabolic capacity, which in turn contributed to a more sustained neuroprotective response to BCAS and HFF, ultimately resulting in greater preservation of cognitive function.

## Discussion

In the present study, we found that IH perconditioning can effectively improve microcirculation and increase CBF in cognition-related brain regions, and enhanced cognitive function in our VCID mouse model. Additionally, IH perconditioning notably ameliorated cognitive impairment in mice following combined BCAS surgery and HFF feeding, even without affecting the associated increases in body weight, blood glucose, and blood lipids (Wu et al., 2022). Finally, we hypothesize that IH-induced CBF improvement enhances mitochondrial metabolic capacity, leading to sustained neuroprotection and preservation of cognitive function in response to BCAS and HFF.

VCID is the second most frequent dementia syndrome after Alzheimer’s disease, but therapeutic options are limited to symptomatic treatment and risk factor management (Rundek et al., 2022). A more direct and potentially effective strategy is maintenance or restoration of normal CBF, and previous research has indicated that IH can safely stimulate cerebral angiogenesis and augment CBF (Guan et al., 2023b). In fact, induction of vascular remodeling by hypoxia has been studied extensively for decades, but most such studies have focused on continuous hypoxia (Guan et al., 2023a). Although chronic and continuous hypoxia can induce cerebrovascular remodeling and enhance the resistance of mice to ischemia/hypoxia-induced cerebral damage (Boroujerdi and Milner, 2015; Zhang et al., 2020), it is usually accompanied by blood–brain barrier destruction and neuronal damage (Schoch et al., 2002; Bauer et al., 2010; Chen et al., 2024). Furthermore, persistent hypoxia can be distressing and thus difficult to induce in the clinical setting. In comparison, IH is safer and more tolerable (Jung and Mallet, 2018; Su et al., 2022; Yuan et al., 2022). For instance, intermittent hypoxic–hyperoxic training has been shown to improve cognitive function and exercise endurance in older adults depending on its magnitude, duration, and frequency (Bayer et al., 2019; Verge et al., 2024). According to our previous study (Guan et al., 2023a), a magnitude of 6%–11% (10%‒15% O_2_), a duration of 2‒8 hours per day, and a frequency of 3‒15 times per day is a relatively safe and effective regimen. Here, we report that 10 cycles of 5-minute 13% O_2_/5-minute hypoxia daily for 14 consecutive days can significantly enhance cerebral vascular microcirculation in the PFC and hippocampus of both naïve mice and VCID model mice, and this treatment can improve cognitive impairment in VCID mice. Collectively, these findings support IH as a potential therapeutic strategy for patients with VCID.

However, the clinical translation of potential VCID therapies faces considerable challenges (Chamorro et al., 2021; Eren and Yilmaz, 2022; Li et al., 2022), including with respect to the selection and construction of animal models that recapitulate the core aspects of the disease (Duncombe et al., 2017). In our study, BCAS surgery was used to establish a VCID mouse model; however, a drawback of this model is the relatively acute reduction in CBF after the surgery. In mice, the reduction in CBF is slower in the gradual common carotid artery stenosis model compared with the BCAS model. In future studies, this model could be used to more accurately mimic the chronic cerebral hypoperfusion in the human brain caused by aging and cardiovascular risk factors (Hattori et al., 2016). The incidence of VCID gradually increases with age; thus, the disease is usually accompanied by comorbid disorders of aging, most notably metabolic disorders associated with vascular risk factors (van der Flier et al., 2018), which can contribute to the progression of cognitive impairment (Dichgans and Leys, 2017). Therefore, incorporating these risk factors into animal models is essential for improving the clinical translation of intervention measures. In this study, we established a VCID mouse model using surgery and fed these mice a diet rich in saturated fat and fructose. A diet rich in saturated fat, and ensuing high blood lipids, promotes glucose intolerance, obesity, coronary heart disease, and type 2 diabetes, whereas fructose is predominantly metabolized in the liver, where it is rapidly converted into glucose, glycogen, lactate, and fat (Ding et al., 2024). Prolonged high consumption of fructose by rodents can lead to liver and extrahepatic insulin resistance, obesity, type 2 diabetes, and hypertension (Im et al., 2021; Yki-Järvinen et al., 2021; Wu et al., 2022). In the current study, naïve mice fed an HFF diet exhibited elevated body weight, blood sugar, and blood lipids as well as impaired cognition. Although IH did not improve these metabolic abnormalities, it ameliorated the associated CBF and cognitive impairments. However, additional studies are required to investigate other important risk factors, such as age and sex, to further enhance the clinical translatability of our findings.

The mechanism underlying these therapeutic effects of IH also requires considerable additional investigation. Our proteomics analyses identified numerous proteins that were differentially expressed following IH, including several of the mitochondrial respiratory chains such as Ndufa3, Ndufb8, Ndufb9, and Cox6c. In prior studies conducted by our team, it was established that IH can induce cerebral angiogenesis (Guan et al., 2023b). In the present study, we observed that IH effectively enhances the vascular network in brain regions associated with cognition. This improvement in the cerebral vascular network is a key factor contributing to the increased CBF observed in VCID mice. We hypothesize that the regulation of energy-related proteins and neuronal protection are consequences of the enhanced CBF induced by IH. Thus, neuronal protection may be considered a downstream effect of improved cerebral perfusion.

This study has several limitations. First, although an HFF diet was included, other clinically prevalent risk factors, such as age and sex, were not accounted for in the experimental design. Second, although we hypothesize that IH-induced improvements in CBF enhance mitochondrial metabolic capacity, thereby promoting sustained neuroprotection and preservation of cognitive function in response to BCAS and an HFF diet, further experimental validation is required to substantiate this mechanism. Lastly, the hypoxia model was developed using mice, and additional investigations are necessary to optimize hypoxic protocols such that they are suitable for clinical translation in patients.

In summary, we demonstrate that IH can improve CBF, cerebrovascular microcirculation in the PFC and hippocampus, and cognitive function in a mouse model of VCID, including comorbid risk factors. The results of this study support IH as a potential therapeutic strategy for VCID and identify numerous molecular targets for clinical manipulation.

## Additional files:

***Additional file 1:*** *Fluoro-Jade B staining.*

Additional file 1

***Additional file 2:*** *Microglia staining.*

Additional file 2

***Additional Figure 1*** *: Intermittent hypoxia (IH) does not alter the fundamental characteristics of microvessels.*

Additional Figure 1 Intermittent hypoxia (IH) does not alter the fundamental characteristics of microvessels.
(A) Representative images of brain tissue slices and the whole brain before and after tissue clearing and imaging. (B–G) Quantification of vessel mean diameter in the prefrontal cortex (PFC) (B) and hippocampus (E) (*n* = 4 or 5 mice per group), vessel straightness in the PFC (C) and hippocampus (F) (*n* = 4 or 5 mice group), and vessel resistance in the PFC (D) and hippocampus (G) (*n* = 4 mice per group) of IH group mice and controls. Results are expressed as mean ± standard error of the mean. Con: Control group.

***Additional Figure 2:*** *Intermittent hypoxia (IH) reduces neuronal damage and inflammatory cell infiltration in cognition-related brain areas of vascular cognitive impairment and dementia model mice.*

Additional Figure 2 Intermittent hypoxia (IH) reduces neuronal damage and inflammatory cell infiltration in cognition-related brain areas of vascular cognitive impairment and dementia model mice.
(A–F) Quantification of vessel mean diameter in the prefrontal cortex (PFC) (A) and hippocampus (D) (*n* = 4–6 mice per group), vessel resistance in the PFC (B) and hippocampus (E) (*n* = 4–6 mice group), and vessel straightness in the PFC (C) and hippocampus (F) (*n* = 4–6 mice per group) of sham group mice, bilateral common artery stenosis (BCAS) group mice, and BCAS + IH group mice. (G) Nissl staining of mouse hippocampus. Compared with the sham group, the BCAS group showed a reduction in the number of neurons and disorganized neuronal arrangement in the hippocampal cornu ammonis and dentate gyrus (DG) regions, whereas this phenomenon was improved in the BCAS + IH group. Scale bars: 50 μm (upper), 200 μm (lower). (H) Fluoro-Jade B (FJB) staining of the mouse hippocampal DG region. Compared with the sham group, the BCAS group exhibited neurodegenerative changes in the PFC, whereas these changes were alleviated in the BCAS + IH group. Scale bars: 50 μm. (I) FJB staining of mouse PFC. Compared with the sham group, the BCAS group exhibited neurodegenerative changes in the hippocampal DG region, whereas these changes were mitigated in the BCAS + IH group. Scale bars: 50 μm. (J) Mouse PFC ionized calcium binding adaptor molecule 1 (Iba-1) staining, with activated microglial cells indicated by white arrows. Red color (stained by Alexa Fluor 594) corresponds to Iba-1, and blue color corresponds to 4’,6-diamidino-2-phenylindole (DAPI). Compared with the sham group, the BCAS group showed an increase in the proportion of activated microglia in the PFC, whereas this phenomenon was improved in the BCAS + IH group. Scale bars: 15 μm. (K) Iba-1 staining in the mouse hippocampal DG region. Red color (stained by Alexa Fluor 594) corresponds to Iba-1, and blue color corresponds to DAPI. Compared with the sham group, there was a higher proportion of activated microglia in the hippocampal DG region in the BCAS group. This increase was reduced in the BCAS + IH group. Scale bars: 30 μm. All results are expressed as mean ± standard error of the mean. One-way analysis of variance followed by Tukey’s post hoc test was used in A–F. BCAS + IH: Bilateral carotid artery stenosis combined with intermittent hypoxia perconditioning group; sham: sham surgery group.

***Additional Figure 3:*** *Effects of bilateral common carotid artery stenosis (BCAS), high-fat high-fructose (HFF) diet, and subsequent intermittent hypoxia (IH) intervention on blood cell composition, lipid levels, and neurodegeneration in the hippocampus and prefrontal cortex of mice.*

Additional Figure 3 Effects of bilateral common carotid artery stenosis (BCAS), high-fat, high-fructose (HFF) diet, and subsequent intermittent hypoxia (IH) intervention on blood cell composition, lipid levels, and neurodegeneration in the hippocampus and prefrontal cortex (PFC) of mice.
(A) Mouse white blood cell (WBC; leukocyte) count. (B) Mouse blood neutrophil count. (C) Mouse blood hemoglobin assay. (D) Mouse red blood cell (RBC; erythrocyte) count. (E) Mouse blood monocyte count. (F) Mouse blood lymphocyte count. (G) Mouse blood basophil count. (H) Mouse blood eosinophil count. (I) Determination of mouse serum high-density lipoprotein (HDL) level. (J) Determination of mouse serum low-density lipoprotein (LDL) level. (K) Determination of mouse serum cholesterol level. (L) Determination of mouse serum triglyceride level. (M) Fluoro-Jade B (FJB) staining of the mouse hippocampal dentate gyrus (DG) region. Neurodegenerative changes occurred in the PFC in both the HFF group and the HFF + BCAS group, whereas these changes were mitigated in the HFF + BCAS + IH group. Scale bars: 50 μm. (N) FJB staining of mouse PFC. Neurodegenerative changes were observed in the DG region of the hippocampus in both the HFF and HFF + BCAS groups, whereas these changes were alleviated in the HFF + BCAS + IH group. Scale bars: 50 μm. All results are expressed as mean ± standard error of the mean. Two-way analysis of variance followed by Sidak’s post hoc test was used in A–L. ^*^P < 0.05, ^**^P < 0.01, ^****^P < 0.001. Bas: Basophil; BCAS + IH: bilateral carotid artery stenosis combined with intermittent hypoxia perconditioning group; CHO: cholesterol; Eos: eosinophil; HFF + BCAS: HFF diet combined with bilateral carotid artery stenosis group; HFF + BCAS + IH: HFF diet combined with bilateral carotid artery stenosis and intermittent hypoxia intervention group; HGB: hemoglobin; Lym: lymphocyte; Mon: monocyte; ND: normal diet group; Neu: neutrophil; sham: sham surgery group; TG: triglyceride.

## Data Availability

*All data relevant to the study are included in the article or uploaded as Additional files*.
