# Supplementary figures and images for: Intermittent hypoxic perconditioning improves cognitive function in a mouse model of vascular cognitive impairment and dementia with comorbidities by recovering cerebral blood flow
Source: Neural Regen Res. 2025 Jan 29;21(6):2415–24. doi: 10.4103/NRR.NRR-D-24-00716 (PMC13211803; doi:10.4103/NRR.NRR-D-24-00716)

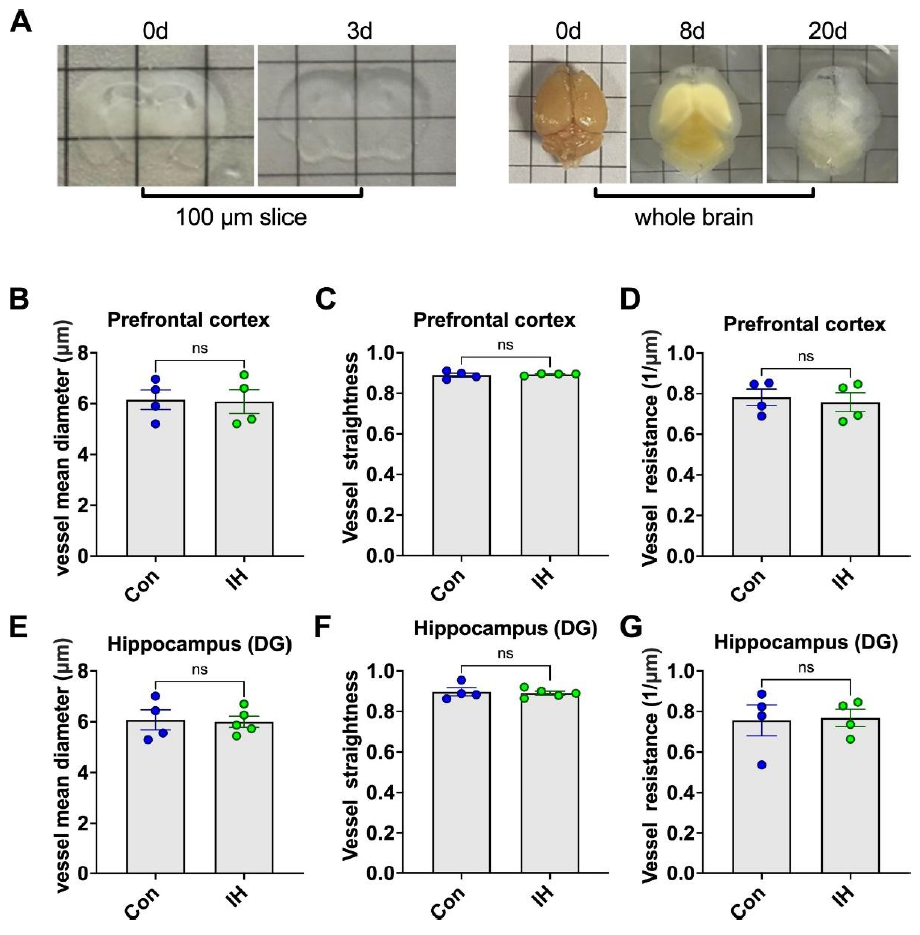

Supplement: Supplementary file 1 [file NRR-21-2415_Suppl1.tif]

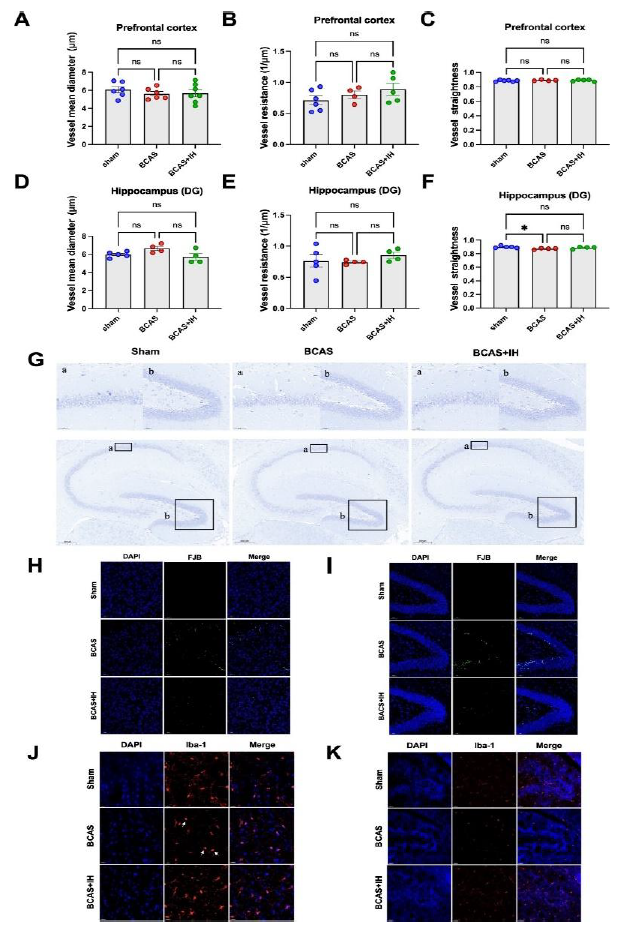

Supplement: Supplementary file 2 [file NRR-21-2415_Suppl2.tif]

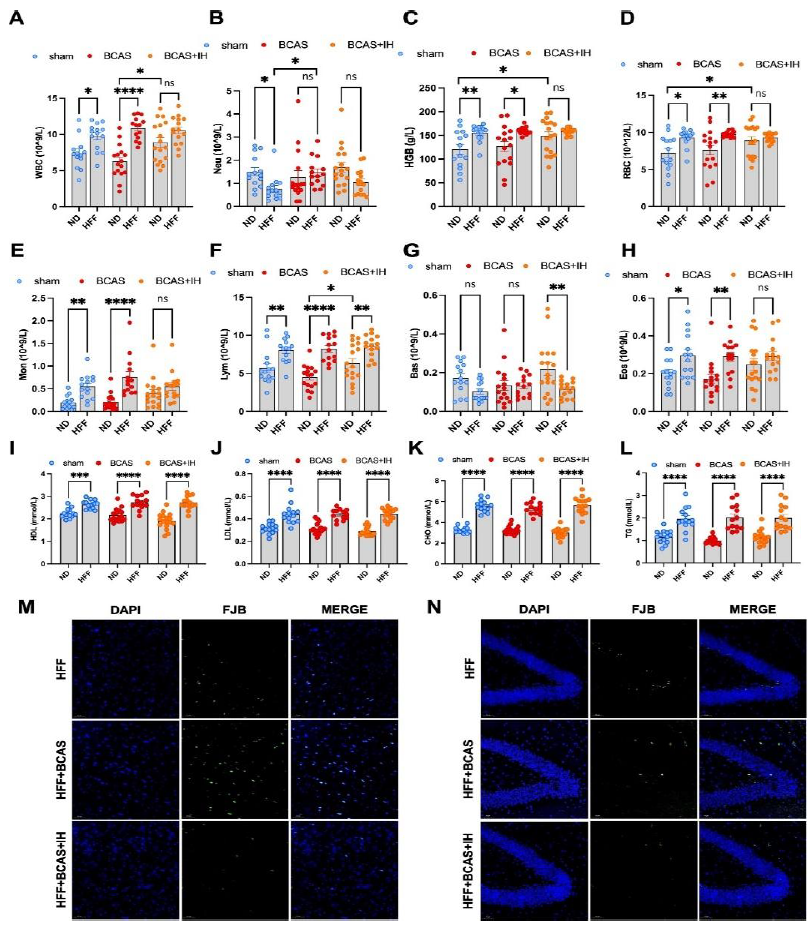

Supplement: Supplementary file 3 [file NRR-21-2415_Suppl3.tif]
