# Supplementary material for: Intermittent hypoxic perconditioning improves cognitive function in a mouse model of vascular cognitive impairment and dementia with comorbidities by recovering cerebral blood flow
Source: Neural Regen Res. 2025 Jan 29;21(6):2415–24. doi: 10.4103/NRR.NRR-D-24-00716 (PMC13211803; doi:10.4103/NRR.NRR-D-24-00716)
Supplement: Supplementary file 4 [file NRR-21-2415_Suppl1.pdf]

**Additional file 1:**

**Fluoro-Jade B staining**

Mouse brain tissue sections were immersed in 100% ethanol for 3 minutes, followed by 70% ethanol for 1 minute, $ddH_2O$ for 1 minute, and 0.006% potassium permanganate for 15 minutes. After rinsing in $ddH_2O$ for 1 minute, the sections were incubated in 0.001% Fluoro-Jade B (Cell Signaling Technology, Beverly, MA, USA) staining solution for 30 minutes, immersed in xylene, dried, and sealed with an anti-quenching sealer containing 4’,6-diamidino-2-phenylindole.
