# Supplementary material for: Intermittent hypoxic perconditioning improves cognitive function in a mouse model of vascular cognitive impairment and dementia with comorbidities by recovering cerebral blood flow
Source: Neural Regen Res. 2025 Jan 29;21(6):2415–24. doi: 10.4103/NRR.NRR-D-24-00716 (PMC13211803; doi:10.4103/NRR.NRR-D-24-00716)
Supplement: Supplementary file 5 [file NRR-21-2415_Suppl2.pdf]

**Additional file 2:**

**Microglia staining**

Mice were anesthetized and then transcardially perfused with cold saline and 4% paraformaldehyde, and the brains were removed. After gradient dehydration of the brains in 20%–30% sucrose, 40-μm coronal sections were made with a cryostat. After routine antigen retrieval, permeabilizing, and blocking, the sections were incubated with anti-ionized calcium binding adaptor molecule 1 antibody (rabbit, 1:500, Cat# 019-19741, RRID: AB_839504; Wako, Osaka, Japan) overnight at 4°C. The sections were then incubated with Alexa Fluor 594-Goat anti-Rabbit IgG (1:500, Jackson ImmunoResearch Labs, West Grove, PA, USA, Cat# A11037, RRID: AB_2534095) at 20-25°C for 1 hour and sealed with an anti-quenching sealer containing 4',6-diamidino-2-phenylindole.
